# The diagnostic value of fine needle aspiration in comparison with frozen section in thyroid nodules: A 20-year study

**DOI:** 10.22088/cjim.8.4.301

**Published:** 2017

**Authors:** Sepideh Siadati, Seyed Mozafar Rabiee, Ebrahim Alijanpour, Mohammad Ali Bayani, Novin Nikbakhsh

**Affiliations:** 1 Cancer Research Center, Health Research Institute, Babol University of Medical Sciences, Babol, Iran.; 2 Department. of Anesthesiology, Babol University of Medical Sciences, Babol, Iran.; 3 Social Determinants of Health (SDH) Research Center, Health Reserch Institute, Babol University of Medical Sciences, Babol, Iran.

**Keywords:** Frozen section, Cytology, Fine needle aspiration, Thyroid nodules.

## Abstract

**Background:**

Fine needle aspiration (FNA) is the most important method in the diagnosis of thyroid nodules before surgery. Recently, the efficiency of FNA in thyroid nodule management has been debatable. On the other hand, intraoperative frozen section (FS) has been used to confirm the diagnosis of FNA and select the proper surgical approach. In this regard, the present study aimed to assess the diagnostic value of FNA as compared to FS in the diagnosis of thyroid nodules.

**Methods:**

This retrospective study was performed on 69 patients with FNA and FS and histopathological examination from 1993 to 2014 in Babol, northern Iran. FNA was classified into 5 groups: benign (colloid goiter), lymphocytic thyroiditis, follicular lesions, suspicious and malignant, and FS was classified as after benign or malignant. The results of both methods were compared with each other.

**Results:**

This retrospective study was performed on 69 patients with FNA and FS and histopathological examination from 1993 to 2014 in Babol, northern Iran. FNA was classified into 5 groups: benign (colloid goiter), lymphocytic thyroiditis, follicular lesions, suspicious and malignant, and FS was classified as after benign or malignant. The results of both methods were compared with each other.

**Conclusion:**

FNA was considered as a simple, less invasive and cost effective method with fewer side effects for evaluation of thyroid nodules. Particulary it had a high sensitivity and specificity in experienced and skilled hands.

**T**hyroid nodules are one of the most common clinical problems and their prevalence was reported 5-10% in routine autopsy reports (1). In one study in Tehran, Iran, the prevalence of thyroid nodules was 3% and 8.3% in men and women, respectively (2). They were important in terms of probability of malignancy. Thyroid gland neoplasm represents the most common endocrine malignancy and 5-10% of the thyroid nodules are malignant (3). Regarding this matter, several measures have been taken to provide presurgery differentiation between benign and malignant nodules (4). The definite diagnosis of these nodules with the use of some diagnostic methods like sonography and radioisotope scanning could not be possible (5). Fine needle aspiration (FNA) is the most cost effective and safest primary diagnostic method in presurgical assessment of thyroid nodules. One of the diagnostic problems of FNA is the undetermined significance and suspicious results obtained in 15-30% of cases (6). Most surgeons prefer intraoperative FS as a guide to determine the extension of surgery (7). 

Considering the role of FS in fast intraoperative differentiated diagnosis of benign or malignant thyroid lesions and selection of the best surgical approach, leads to a decrease in invasive surgical procedures and establishing a less invasive approach. On the other hand, it contributes to a mutual trust between surgeons and pathologists (8). Therefore, this study aimed to determine the diagnostic value of FNA in comparison with FS in the diagnosis of thyroid nodules.

## Methods

 This retrospective study was performed on 225 patients who underwent thyroidectomy at Shahid Beheshti Hospital of Babol, northern Iran from July 1993 to November 2014. Inclusion criteria were FNA, FS and histopathologic examination performed for each patient. In this regard, inadequate FNA was excluded, therefore its results were divided into five groups: 1) colloid goiter 2) lymphocytic thyroiditis, 3) follicular lesion 4) suspicious for malignancy and 5) malignancy. The histopathologic results were divided into two categories: benign and malignant. Data including age, gender, FNA, FS and histopathologic reports were retrieved from pathology archive. The obtained data were analyzed by SPSS Version 22. Chi-square and the Fisher’s test were performed at the significance level of 0.05. Sensitivity, specificity, positive and negative predictive values and diagnostic accuracy of FNA were evaluated using catmaker software.

## Results

Out of 225 patients submitted for FNA, 69 cases had intraoperative FS. Afterwards, the results were compared with final histopathological examination. Of the 69 patients, 60 (87%) patients were females, and 9 (13%) were males. The results of FNA and FS are shown in table 1. Thirty-four (49.3%) of nodules involved the right lobe, 31(44.9%) in left lobe and 4(5.8%) bilateral.

FNA had the greatest sensitivity in the diagnosis of thyroid nodule and the lowest sensitivity in the diagnosis of lymphocytic thyroiditis. Table 2 showed the diagnostic accuracy of FNA in comparison with FS. 

**Table1:** Frequency of thyroid lesions diagnosed by FNA and FS

** Method** **Results**	** FNA** * **N (%)**	** FS** ** **N (%)**
Colloid goiter	45(65.2)	49(71)
Lymphocytic thyroiditis	3 (4.3)	3(4.3)
Follicular lesions	4(5.8)	4(5.8)
Suspicious for malignancy	17(24.6)	-
Malignancy	-	13(18.8)
Total	69	69

* Fine needle aspiration

** Frozen section

**Table 2:** Diagnostic value of FNA in comparison with FS in thyroid nodule

**Results**	**Sensitivity** **(CI 95%)**	**Specificity** **(CI 95%)**	**PPV ** * **(CI 95%)**	** NPV ** ** **(CI 95%)**	** LR+** *** **(CI 95%)**	**LR-** **(CI 95%)**
Thyroid nodule	84 (73-94)	80 (62-98)	91 (83-99)	67 (48-86)	4.18 (1.73-10.14)	0.20 (0.10-0.40)
Lymphocytic thyroiditis	33 (20-87)	97 (93-100)	33 (20-87)	97 (93-100)	11 (1.34-90.12)	0.69 (0.31-1.53)
Follicular lesions	50 (1-99)	97 (93-100)	50 (1-99)	97 (93-100)	16.25 (1.03-87.18)	0.52 (0.19-1.38)
Malignancy	69 (44-94)	86 (77-95)	53 (29-77)	92 (85-100)	4.85 (2.32-10.13)	0.36 (0.16-0.82)

* Positive predictive values

** Negative predictive values

*** Likelihood ratio

## Discussion

The combined use of radioisotope scanning, FNA and histopathology is the best diagnostic approach (9). The prevalence of palpable thyroid nodules was estimated 5%. Nonetheless with the use of ultrasonography, the 50% of population may have thyroid nodules (10). In this study, based on FNA, 75.3% of lesions were reported benign, which can be compared with studies done by Kumar (88%), Riazi (74.7%) and Bahar (79.8%) (7, 11, 12). As mentioned above, FNA had been considered as a presurgical, golden standard method in the detection of thyroid carcinoma. Nevertheless, in recent years, FNA has become very popular in the evaluation of single thyroid nodules, because it is fast, simple, cost effective and accurate outpatient method with high sensitivity and specificity (6). In the present study, there is a significant female preponderance (87%). This result was in accordance with studies done by Kumar (84%), Riazi (84.3%) and Bahar (89.5%) (7, 11, 12). All these studies showed a significant number of women reported with thyroid nodule. In our study, the most and the least sensitivity of FNA was seen in the diagnosis of thyroid nodule and lymphocytic thyroiditis, respectively. 

Several factors can affect the diagnostic value of FNA in detecting thyroid malignancy including sampling error, heterogencity of the nodule and suboptimal slide preparation. Finally, the experience of physician in the performance of FNA is also an essential factor (6). According to our findings, 34(49.3%) of nodules involved the right lobe, 31(44.9%) left lobe and 4(5.8%) bilateral. In Bahar’s study, most nodules were located in the right lobe (53%), 4.01% in the left lobe, 2.2% bilaterally that was similar to our results (12). Papillary carcinoma with 69% frequency was the most common malignancy in our study which was in consistence with Esteghamati and Nakhjavani with 80% and 70% frequency of papillary carcinoma, respectively (13, 14).

In Taghavi et al.’s study, FNA in the diagnosis of thyroid nodule had the sensitivity, specificity; PPV and NPV of 82.3%, 78.9%, 77.8% and 83.3%, respectively (15). In the study of Mirsadraei et al. in 2007, FNA results had specificity, sensitivity, accuracy, NPV and PPV of 89.5%, 91.5%, 93.7%, 80.9% and 95.5%, respectively (16). Siadati *et al.* reported 60% sensitivity, 96% specificity, 65% PPV and 95% NPV (17). 

Futhermore, Gong *et al*. reported that FNA has an important role in the evaluation and differentiation of benign from malignant nodules (18). The current study confirmed the important role of FNA as a simple, cost effective and noninvasive method and its high sensitivity and specificity if examined by experienced pathologist. 
